# Development of a Soft Sensor Using Machine Learning Algorithms for Predicting the Water Quality of an Onsite Wastewater Treatment System

**DOI:** 10.1021/acsenvironau.2c00072

**Published:** 2023-06-30

**Authors:** Hsiang-Yang Shyu, Cynthia J. Castro, Robert A. Bair, Qing Lu, Daniel H. Yeh

**Affiliations:** Civil & Environmental Engineering, University of South Florida, 4202 E. Fowler Avenue, Tampa, Florida 33620, United States

**Keywords:** onsite wastewater treatment system, machine learning, soft sensor, water quality, wastewater monitoring

## Abstract

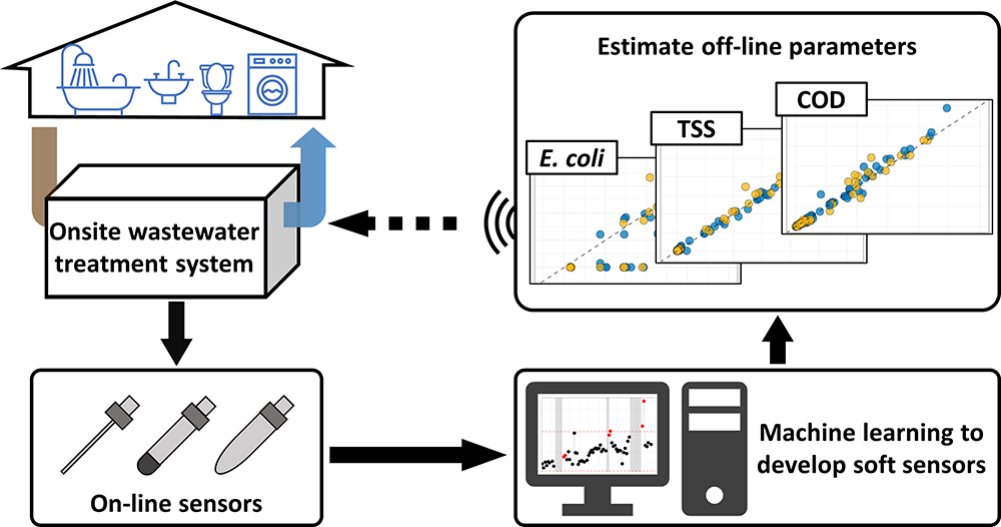

Developing advanced onsite wastewater treatment systems (OWTS) requires accurate and consistent water quality monitoring to evaluate treatment efficiency and ensure regulatory compliance. However, off-line parameters such as chemical oxygen demand (COD), total suspended solids (TSS), and *Escherichia coli* (*E. coli*) require sample collection and time-consuming laboratory analyses that do not provide real-time information of system performance or component failure. While real-time COD analyzers have emerged in recent years, they are not economically viable for onsite systems due to cost and chemical consumables. This study aimed to design and implement a real-time remote monitoring system for OWTS by developing several multi-input and single-output soft sensors. The soft sensor integrates data that can be obtained from well-established in-line sensors to accurately predict key water quality parameters, including COD, TSS, and *E. coli* concentrations. The temporal and spatial water quality data of an existing field-tested OWTS operated for almost two years (*n* = 56 data points) were used to evaluate the prediction performance of four machine learning algorithms. These algorithms, namely, partial least square regression (PLS), support vector regression (SVR), cubist regression (CUB), and quantile regression neural network (QRNN), were chosen as candidate algorithms for their prior application and effectiveness in wastewater treatment predictions. Water quality parameters that can be measured in-line, including turbidity, color, pH, NH_4_^+^, NO_3_^–^, and electrical conductivity, were selected as model inputs for predicting COD, TSS, and *E. coli*. The results revealed that the trained SVR model provided a statistically significant prediction for COD with a mean absolute percentage error (MAPE) of 14.5% and *R*^2^ of 0.96. The CUB model provided the optimal predictive performance for TSS, with a MAPE of 24.8% and *R*^2^ of 0.99. None of the models were able to achieve optimal prediction results for *E. coli*; however, the CUB model performed the best with a MAPE of 71.4% and *R*^2^ of 0.22. Given the large fluctuation in the concentrations of COD, TSS, and *E. coli* within the OWTS wastewater dataset, the proposed soft sensor models adequately predicted COD and TSS, while *E. coli* prediction was comparatively less accurate and requires further improvement. These results indicate that although water quality datasets for the OWTS are relatively small, machine learning-based soft sensors can provide useful predictive estimates of off-line parameters and provide real-time monitoring capabilities that can be used to make adjustments to OWTS operations.

## 1 Introduction

Onsite wastewater treatment systems (OWTSs) serve at least 20% of residences in the United States, and many developing countries rely on onsite systems to an even greater extent.^1^ Traditional OWTSs, such as septic tanks, cesspools, subsurface infiltration systems, aerobic treatment units, and sand filters, have been used as reliable sanitation systems for decades. More recently, advanced treatment technologies, such as electro-oxidation and membrane bioreactors, have been applied as OWTSs.^2^ Although modern OWTSs are highly effective at wastewater treatment, older systems and ones lacking adequate maintenance have been linked to nutrient pollution of ground and surface waters along with pathogen outbreaks.^3−5^ For OWTSs, water quality parameters and pathogen measurements require manual sampling, transport, and lab analyses, which are both costly and time-consuming. Moreover, monitoring multiple sites for these parameters can be expensive, potentially leading to delayed detection of system failure and undetected contamination of the surrounding environment.^6^ It is crucial to develop novel monitoring solutions for OWTSs that enable significant reductions in sampling and analysis costs as well as provide emergency notifications to the responsible parties during performance deficiency events. These steps are needed to ensure that public and environmental health are protected from harmful contaminants.

Most large-scale wastewater treatment plants (WWTPs) have in-line water quality sensors, such as pH, dissolved oxygen (DO), temperature, oxidation–reduction potential (ORP), electrical conductivity (EC), and turbidity to monitor treatment efficacy, stability, and process control and to identify process abnormalities.^7−9^ Advancements in sensor technologies have made the real-time estimation of relevant ions in WWTP processes, such as nitrate (NO_3_^–^) and ammonium (NH_4_^+^), possible with the use of ion-selective electrodes. However, parameters such as chemical oxygen demand (COD), total suspended solids (TSS), and pathogens still lack a reliable and cost-effective in-line sensor equivalent. Although analyzers and spectral absorbance-based instruments for COD and TSS exist, these tools have time delays and require frequent maintenance when exposed to the high loading rates of organics, metals, and salts that make up wastewater.^10−12^ This burden can be eliminated by incorporating additional equipment, such as compressors, that provide automated blasts of compressed air to remove biofilms on the sensor surface.^13,14^ However, most analyzers have limitations on sample concentration and quality. For example, one commercial in-line COD analyzer can only measure a fixed concentration range between 40–500 mg/L and the TSS in the water sample cannot exceed 0.1 g/L.^13^ Due to the high capital, maintenance, and chemical costs associated with in-line analyzers, along with the measurement limitations imposed by interference restrictions, their use is primarily confined to large WWTPs. Consequently, these analyzers become extremely cost-prohibitive for smaller facilities like OWTSs.

Most recently, significant progress has been made in the development of data-driven modeling approaches for wastewater treatment process control and monitoring using artificial intelligence-based methods such as machine learning (ML). Data-driven soft sensors use a combination of real-time data inputs and mathematical models to estimate complex parameters when the correlated parameters can be measured in real-time. Studies have highlighted the potential of soft sensors to predict challenging off-line parameters in WWTPs.^15−18^ These studies include soft sensors for monitoring floc size to control coagulant dosage,^19^ monitoring of *Escherichia coli* (*E. coli*) concentrations,^20^ monitoring the COD and TSS of restaurant effluent,^21^ and predicting treatment efficiency.^22^ However, these studies have all focused on centralized WWTPs, while OWTS has been largely neglected. The challenge of applying soft sensors to OWTS is largely due to the infrequency of data collection. WWTPs have the advantage of frequent data collection, whereas data collection for OWTSs is often infrequent and limited due to financial constraints and challenges in accessing remote locations. OWTSs may be sampled only 1–4 times a year depending on local regulations,^23,24^ while some advanced OWTSs may be sampled only once every five years.^25^ Although there is no minimum dataset size for soft sensor development, large data sets generally enhance the prediction robustness of ML algorithms. In cases where dataset size is limited, the selection of a suitable dataset that encompasses a diverse range of operations and features becomes crucial. This enables the algorithms to capture a wider spectrum of patterns and relationships that may otherwise fall beyond their typical boundaries.^26,27^

In this study, two years of water quality data from an advanced OWTS operating in a South African informal settlement was used to develop data-driven soft sensors. The field trial data were used to train the ML regression algorithms, which were developed to predict off-line water quality parameters, including COD, TSS, and *E. coli*. By estimating these off-line parameters, operators can make predictions of the quality and safety of their effluent and allow for early detection of process abnormalities. The study utilized the water quality parameters from the OWTS dataset to train and evaluate four ML regression models, namely, partial least square regression (PLS), support vector regression (SVR), cubist regression (CUB), and quantile regression neural network (QRNN). The performance of each ML algorithm was evaluated for predicting COD, TSS, and *E. coli* concentrations independently as a multi-input and single-output soft sensor, with one model developed for each parameter. The spatial prediction performance was also assessed across the four treatment processes used within this specific OWTS, including anaerobic digestion, ultrafiltration, adsorption, and electrochlorination. A discussion is presented on the overall performance and limitations of the ML model prediction and gives suggestions to further enhance the prediction power of soft sensors for remote OWTS monitoring.

## 2 Materials and Methods

### 2.1 System Configuration

**Figure 1:**
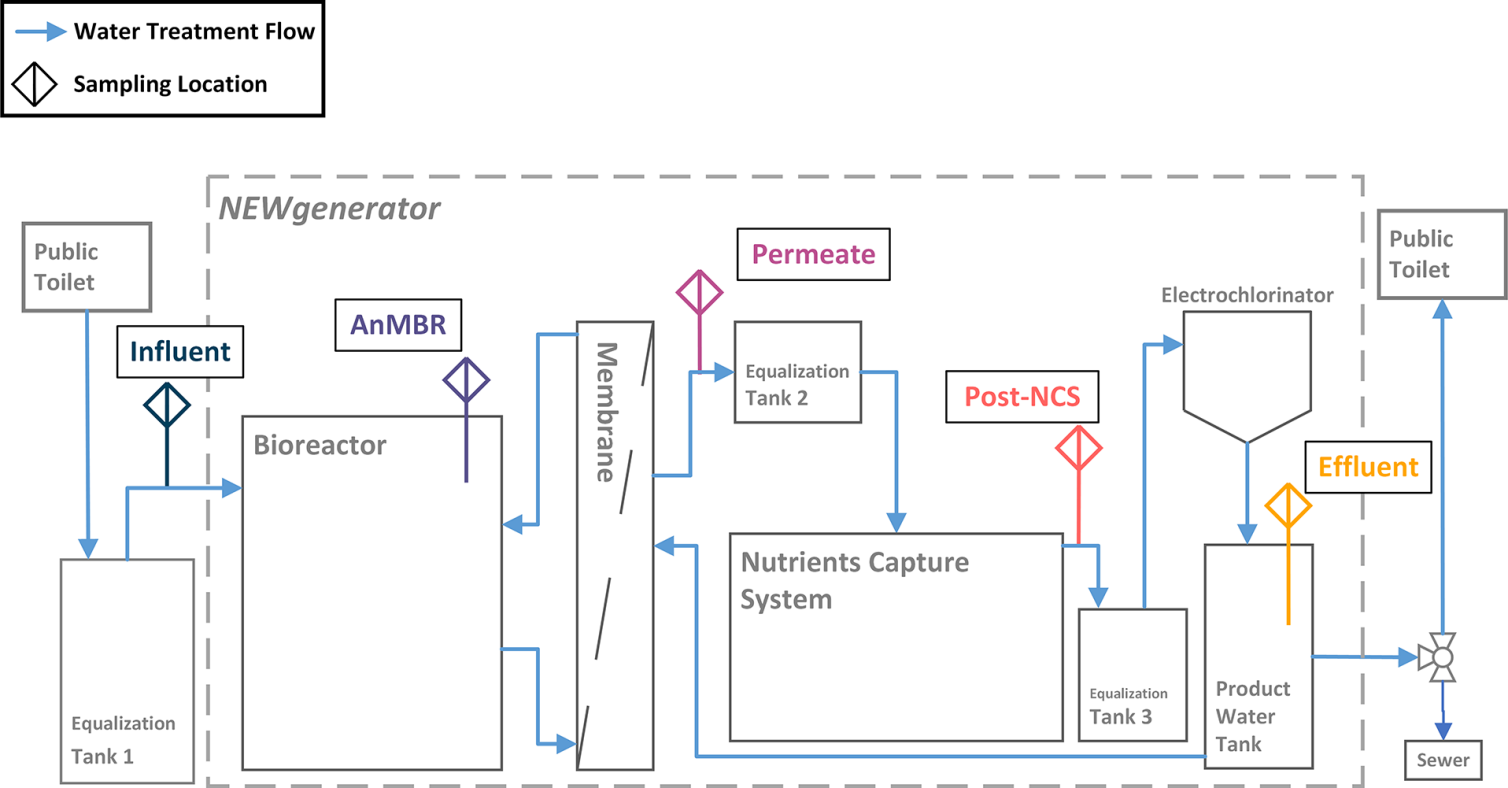
Process schematic for the NEWgenerator and the five sampling points for each treatment step (Influent, AnMBR, Permeate, Post-NCS, and Effluent). The modules inside the dashed line were housed in a shipping container.

The water quality data used in this study was obtained from an existing field-tested OWTS called the NEWgenerator (NG), which is operated at an informal settlement in the eThekwini Municipality of South Africa to treat blackwater and yellow water for almost 2 years. The system design, experimental conditions, operation, and performance have been described by Shyu et al.^28^ and Castro et al.^29^ In summary, the system comprised of three main treatment processes: an anaerobic membrane bioreactor (AnMBR), a nutrient capture system (NCS), and an electrochlorinator (Figure 1).

### 2.2 Field Water Quality Data Collection

Water quality results were sampled from the five sampling points of the NG, including system influent (Inf), AnMBR, permeate (Perm), post-NCS (P-NCS), and effluent (Eff). Each sampling point was sampled on a weekly basis, unless otherwise noted (Table S1). During the field test, sampling and analysis followed standard methods described by the American Public Health Association.^30^ Seven water quality parameters conventionally available for in-line measurements were selected for the development of three soft sensor models to predict TSS, COD, and *E. coli*. The water quality parameters used included NH_4_^+^, NO_3_^–^, pH, EC, turbidity, color, and temperature. The parameters COD, TSS, NH_4_^+^, and NO_3_^–^, were measured according to Methods 5220D, 2540D, 4500-nh3, and 4500-no3, respectively. *E. coli* was measured using the Quanti-Tray system (IDEXX, Quanti-Tray 2000, USA) with a detection limit of 1 MPN/100 mL. As most of the *E. coli* data for the Eff and P-NCS samples were below the detection limit (BDL), omitting these observations would reduce the prediction accuracy of the soft sensors. Therefore, a substitution method was used to input the BDL data into the regression model. This method replaced the BDL data with a value of 1 MPN/100 mL, which in practice has no significant difference with the true unknown values. The pH and EC were measured with a multi-parameter probe (Hach, HQ40D, USA). The color and turbidity of the water samples were measured with a colorimeter (Hach, Color Test Kit Model CO-1, USA) and a turbidimeter (Hach, 2100Q IS, USA). Temperature was measured with an in-line sensor (HOBO, S-TMB-M002, USA) and included the daily average ambient air temperature and the daily average temperature in the AnMBR.

### 2.3 Statistical Analyses for Water Quality Data

During the field trial, the NG did not operate continuously due to maintenance events and holidays that caused some system shutdowns and restarts. The summer vacations during December of 2018 and 2019 and the NCS maintenance performed in August 2019 and January 2020 caused prolonged shutdowns of the NG. A detailed description of all events can be found in Castro et al.^29^ In total, water quality data for 56 weeks were available for model development and testing. Welch’s *t* test was used to identify non-steady state water quality data of individual parameters by selecting two consecutive data points after each significant restart event. In addition, a *z*-score analysis on the daily water quality parameters was used to detect any non-steady state conditions that may have resulted from the restart events. The *z*-score is a statistical technique commonly used to identify data outliers by measuring the differences between the standard deviations and observations from the mean of the distribution.^31^ The formula for this calculation is as follows:
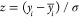
1where *y_i_* is the observed value, *y_i_®* is the mean of observed values, and σ is the standard deviation of observed values.

### 2.4 Machine Learning Algorithms

Four ML algorithms were selected for developing the soft sensors, including PLS, SVR, CUB, and QRNN. These algorithms have successfully been implemented in wastewater treatment applications with a track record of high prediction accuracy.^26,32−34^ However, their selection for this study was based on their exceptional performance across a wide range of data scenarios, their ability to accommodate diverse feature distributions, and their capacity to handle complex correlations. These characteristics make them potentially suitable for application in the context of OWTS. All models were trained, evaluated, and validated in R 4.2.0.^35^ The four ML algorithms were implemented using the ‘caret’ package.^36^ The PLS (package: ‘pls’) is a linear regression model based on the dimension reduction method^37^ and was selected as an advanced linear model to compare the prediction performance with other models. The hyperparameter, principal components (ncomp), was tuned to improve the prediction and avoid overfitting. The SVR (package: ‘svmRadial’) is a statistical learning-based approach that transforms the original variables into a high-dimensional space and separates them by defining a hyperplane. The radial basis function kernel for SVR was selected in this study due to its ability to model complex non-linear relationships between parameters and its flexibility in optimizing the hyperparameters.^38^ Two hyperparameters were used, inducing the kernel (sigma) and class weights (cost) to control the shape of the decision boundary and balance the margin-maximizing and error-minimizing objectives. The CUB (package: ‘cubist’), which is an extension of the decision trees, works by recursively partitioning the input data into regions, then fitting a linear model to the data within each region. The final outcome of CUB is obtained by combining the predictions of the individual linear models.^39^ The CUB model was selected as it can represent non-linear relationships between variables. This study optimized the hyperparameters in the CUB model by including iterative model trees created in sequence (committees) and nearest neighbors to both improve the model accuracy and to avoid overfitting the model. The QRNN (package: ‘qrnn’) is an extension of a neural network specifically designed for linear quantile regression. The QRNN model can effectively capture non-linear relationships and non-normally distributed parameters, allowing it to estimate different quantiles of the output distribution and provide more accurate predictions. The ability to model different quantiles also enables the QRNN to capture the variability in the data.^33^ Due to the size limitation of the trained dataset, two hyperparameters, hidden-layer node (n.hidden) and predictive density from quantiles (penalty), were tuned to avoid model overfitting.

### 2.5 Soft Sensor Model Development

**Figure 2:**
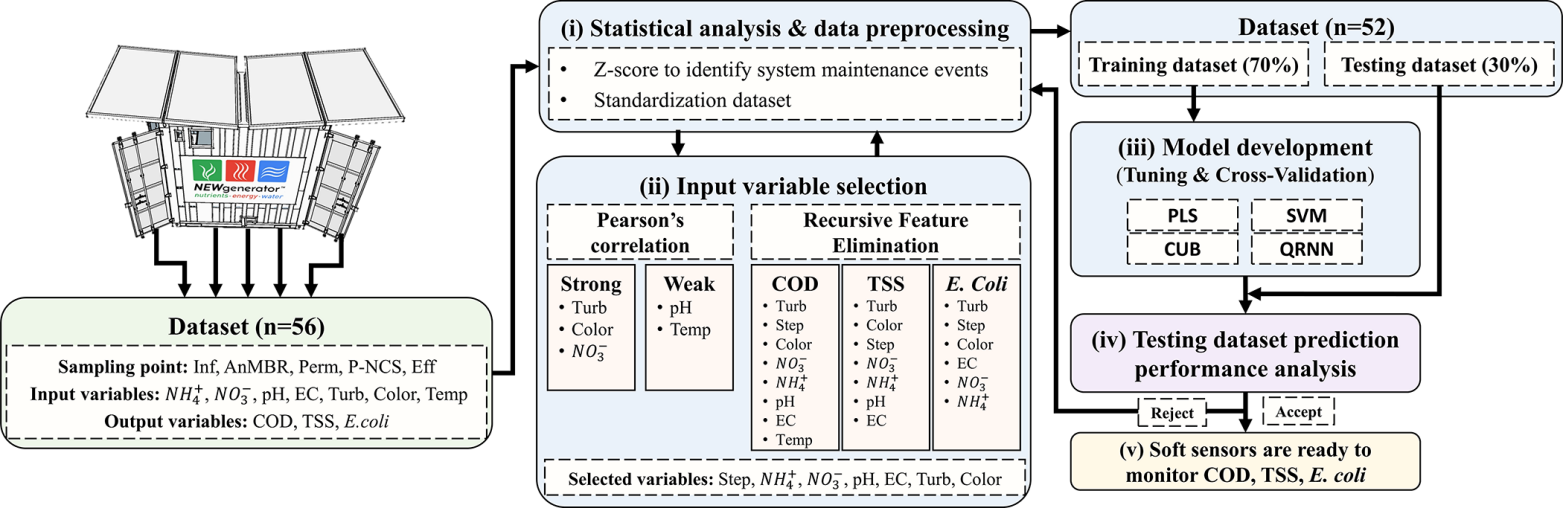
Soft sensor development framework was comprised by the following five steps: (i) statistical analysis and dataset preprocessing; (ii) model input selection using the Pearson’s correlation coefficient and Recursive Feature Elimination method (RFE); (iii) model development, including cross-validation for metrics tuning and model calibration; (iv) model testing for ML algorithms to compare the prediction accuracy; and (v) final soft sensor models for COD, TSS, and *E. coli* prediction.

The dataset (*n* = 56) with eight input parameters (sampling points, NH_4_^+^, NO_3_^–^, pH, EC, turbidity, color, and temperature) and three output parameters (COD, TSS, and *E. coli*) were used to train the soft sensor models for predicting the water quality throughout the system. The framework for developing the three models (Figure 2) follows five main steps: (i) statistical analysis and dataset preprocessing; (ii) model input selection using the Pearson’s correlation coefficient and Recursive Feature Elimination method (RFE); (iii) model development including cross-validation for metrics tuning and model calibration; (iv) model testing for ML algorithms to compare the models’ prediction accuracy; and (v) final soft sensor models for COD, TSS, and *E. coli* prediction.

Considering the wide range of input and output variables in the dataset (Table S1), a standardization procedure was conducted before fitting the ML algorithm to reduce noise and increase the comparability of input variables. This involved centering and scaling the variables. Centering subtracted the mean from each data point, resulting in variables with a mean of zero. Scaling the variables involved dividing each data point by the standard deviation of the variable, which ensured that all variables were on the same scale. This standardization preprocessing made the variables comparable and further reduced the impact of differences in parameter weights on the performance of the ML algorithms.

Pearson’s correlation coefficient with significance testing and RFE technique were used to identify correlations between the variables during the variable selection step. This was performed to detect and remove redundancy and avoid overfitting issues before training the ML algorithms.^40^ The RFE is a feature selection algorithm that selects and iteratively tests the input variables of different datasets by training in the ML algorithm to remove redundant parameters and identify the most relevant variables for predicting the output variables.^41^ This makes the RFE better enabling to accurately define non-linear correlations than the Pearson’s correlation coefficient.

In the soft sensor development process, 70% of the dataset was randomly selected and used to train the models, while the remaining 30% was used to test the trained models.

In model cross-validation and hyperparameter tuning, considering the cost of increased computational time and the slowdown in the downward trend of RMSE with k, a stratified 15-fold cross-validation resampling method was selected and employed to predict and enhance the prediction performance of each model (Figure S1). This approach randomly divides the training dataset into 15 partitions, fits the model to a dataset consisting of 14 of the original 15 partitions, and uses the rest for verification to estimate the error and determine the performance of each model when fitting the training dataset.^42^ The ‘expand.grid’ function in R was used to grid search and determine the best hyperparameters for specific algorithms during model training process.^35^

To assess the regression model performance, standard residuals, root mean square error (RMSE) (eq 2), coefficient of determination (*R*^2^) (eq 3), and mean absolute percentage error (MAPE) (eq 4) were selected to quantifiably analyze the prediction performance of the models.^43^
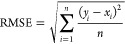
2
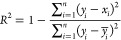
3
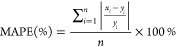
4where *y_i_* is the observed value, *x_i_* is the predicted value by the model, *y_i_®* is the mean of all observed values, and *n* is the number of samples.

To observe and prevent overfitting of the model, the *R*^2^ and scatter plots were used to compare the measured and predicted values of the training and testing datasets for each model. The scatter plots depict the predicted values plotted against the true values, with an ideal scenario exhibiting a 1:1 alignment along a best-fit line. Additionally, RMSE and MAPE were used to evaluate the final soft sensor model. Both RMSE and MAPE metrics are commonly used to assess the performance of the models but differ in how they weight errors. While RMSE emphasizes larger errors, MAPE gives equal weight to all errors, regardless of their magnitude.^20^ As the models were used to predict water quality parameters, which can impact both human and environmental health, it is crucial to minimize large errors. Therefore, the prediction with the lowest RMSE was given priority as the optimal model. In addition, to ensure the usefulness of the soft sensors, a MAPE below 25% was considered acceptable for use.

## 3 Results

### 3.1 Field Data Preprocessing

**Figure 3:**
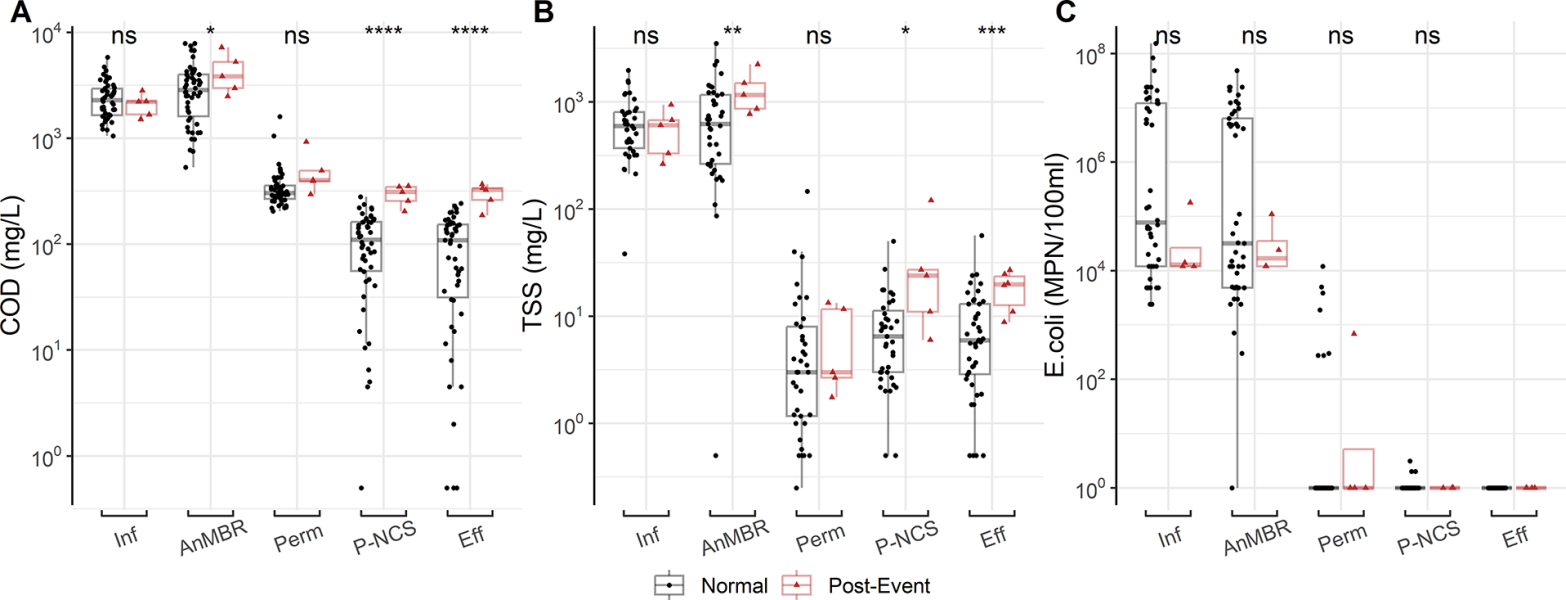
Water quality characteristics for (A) COD, (B) TSS, and (C) *E. coli* in each sampling point and the post-restart event *t* test. The five sampling points were influent (Inf), AnMBR, permeate (Perm), post-NCS (P-NCS), and effluent (Eff). The lines in the whisker plot indicate medians, boxes 25th and 75th percentiles. A paired samples *t* test was used to compare measurements between the post-restart events and the normal operations water quality characteristics. “ns” = *p* > 0.05, “*” = *p* ≤ 0.05, “**” = *p* ≤ 0.01, “***” = *p* ≤ 0.001, “****” = *p* ≤ 0.0001.

Three major restart events occurred during the field trial that caused abnormal spiking of several water quality parameters.^28^ Two of the NCS’s maintenance events had a significant impact on system performance as indicated by substantial peaks in the *z*-score (Figure S2). The system returned to steady state operation after two weeks, as indicated in the *z*-score analysis. These two events created significant data outliers, which are known to negatively affect the predictive performance.^44^ The difference between steady state operation and post-event operation was further confirmed by Welch’s *t* test (Figure 3).

Welch’s *t* test revealed significant differences in the temporal COD and TSS concentrations across several sampling points, including the P-NCS and Eff samples where adsorption and electrochlorination were the main treatment steps, respectively. For P-NCS and Eff samples, a notable distinction was observed between the COD levels during normal operation and post-events (*p* ≤ 0.001 and *p* ≤ 0.01). This disparity was attributed to biofilm detachment within the NCS during periods of dormancy and washout of COD from the AnMBR upon system restart. Similarly, the TSS in the Eff also showed significant differences after the events (*p* ≤ 0.01) likely due to washout of biological growth from the NCS during the system restart. To obtain the best prediction for normal operating conditions, two weeks of water quality data after the two NCS maintenance events were removed from further evaluation, decreasing the total dataset from 56 to 52 points.

### 3.2 Input Selection and Model Development

**Figure 4:**
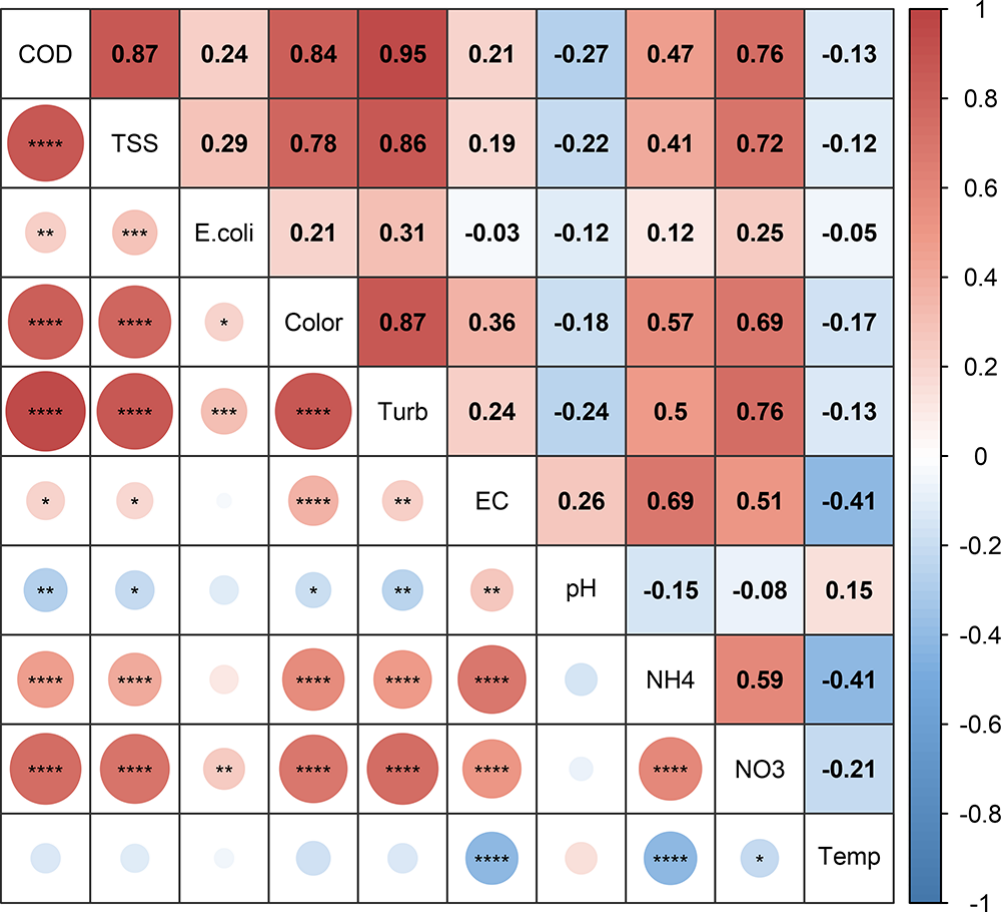
Heat map of the Pearson correlation coefficient and Welch’s *t* test *p*-value between each variable. Red colors denote a positive correlation, whereas blue represents a negative correlation. “*” = *p* ≤ 0.05, “**” = *p* ≤ 0.01, “***” = *p* ≤ 0.001, “****” = *p* ≤ 0.0001.

The pH and temperature variables showed a weak negative correlation with the three desired output variables, COD, TSS, and *E. coli*, in the Pearson’s correlation analysis (Figure 4). The correlation coefficients between temperature and COD, TSS, and *E. coli* were −0.13, −0.12, and −0.05, respectively, while for pH, they were −0.27, −0.22, and −0.12. The results showed negative correlations between temperature and pH and the output parameters. However, these results were not statistically significant (*p* > 0.05). The low correlations observed between temperature and the output variables may be the result of using the average daily temperature, which does not accurately represent the specific temperature at the time of sample collection. Surprisingly, the low correlation of the output parameters with pH was unexpected, considering that fluctuations in pH are known to affect biological treatment systems, particularly anaerobic digestion.^45^ However, the lack of significant correlation with pH was generally attributed to a minimal fluctuation in pH (Table S1).

On the other hand, turbidity, color, and NO_3_^–^ showed a strong positive correlation (coefficients >0.70) with the three output variables. The strong correlation (*p* ≤ 0.0001) between the output parameters and turbidity and color was expected as high values of either parameter often indicating contaminated water samples.^20,46^ With regards to NO_3_^–^, its strong correlation with the three output variables may be attributed to the carbon and nitrogen ratios necessary for biological nitrogen cycling in the NG system. Changes in NO_3_^–^ concentrations are expected to follow the rates of nitrification and denitrification occurring across the treatment train. These processes directly affect the COD and TSS in the water sample as denitrification utilizes COD as a source.^45^

The RFE feature selection method was applied to each of the four ML algorithms to identify and rank important input variables in predicting COD, TSS, or *E. coli* (Table 1 and Figure S3). The results of the RFE were similar to Pearson’s correlation analysis. Temperature was the least selected variable among all ML algorithms. The PLS was the only algorithm that identified temperature as a variable for predicting COD and *E. coli*, although it was relatively insignificant when compared to other variables. The second least selected variable was pH. When predicting *E. coli* concentrations, none of the algorithms selected pH as an important input variable.

**Table 1:** RFE Results for the Input Variables for Predicting COD, TSS, and *E. coli*^a^

variable (unit)	model structure	sampling point	color (Pt/Co)	turbidity (NTU)	pH	EC (mS/cm)	NH_4_^+^ (mg/L)	NO_3_^–^ (mg/L)	temperature (°C)
COD (mg/L)	PLS	1	4	5	3	–	–	2	6
	SVR	4	2	1	6	7	5	3	8
	CUB	2	–	1	7	3	5	4	6
	QRNN	4	2	1	6	–	5	3	–
TSS (mg/L)	PLS	–	1	2	–	3	4	5	–
	SVR	4	2	1	6	7	5	3	–
	CUB	6	4	1	7	5	3	2	–
	QRNN	4	2	1	7	6	5	3	–
*E. coli* (MPN/100 mL)	PLS	6	1	2	–	3	4	5	7
	SVR	–	3	2	–	1	5	4	–
	CUB	–	2	1	–	3	4	5	–
	QRNN	5	3	2	–	1	–	4	–

a The values represent the variable in the order of importance for the specific ML algorithm. The “–” in the table represents a variable that was not selected as an input variable while getting the lowest RMSE in each algorithm REF analysis.

The RFE results showed that EC was selected last by most ML algorithms when predicting COD and TSS. These results agreed with the Pearson’s correlation analysis which observed correlation coefficients of less than 0.3 (*p* ≤ 0.01) between EC and all output variables. However, RFE analysis identified EC as an important variable for predicting *E. coli* in all ML algorithms. The correlation found between EC and *E. coli* offers similar results to the Lambrou et al.^15,100^ study, which used low-cost sensors to track *E. coli* in drinking water. In this study, EC significantly changed when *E. coli* was detected and may have been caused by the fluctuating chlorine concentration in the municipal water supply. A higher concentration of chlorine has higher EC and can effectively reduce *E. coli* in water and even wastewater, and this correlation may be non-linear, which was not observed in the Pearson’s correlation.

Although the Pearson correlation results showed a weak negative linear correlation between pH and the three output variables, pH still played an important role in predicting COD and TSS, as indicated by the RFE analysis. Therefore, temperature, which had a poor correlation with all three output variables, was not selected as an input variable for model development. The final selected input variables for developing the soft sensor included the sampling step, turbidity, color, pH, NH_4_^+^, NO_3_^–^, and EC.

### 3.3 Model Prediction of the Field Water Quality

**Figure 5:**
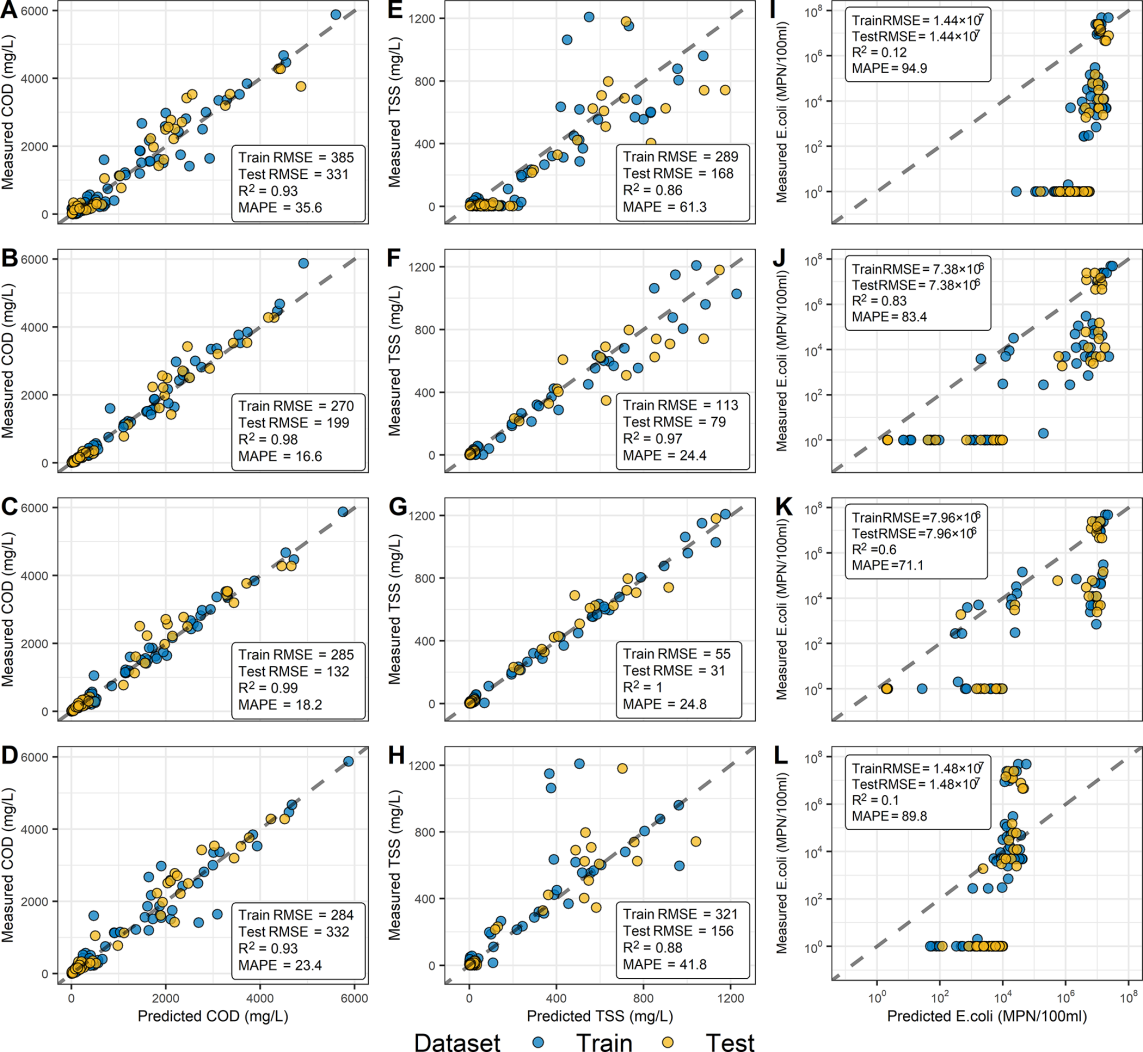
Model prediction for COD, TSS, and *E. coli*. (A–D) PLS, SVR, CUB, and QRNN for COD; (E-H) PLS, SVR, CUB, and QRNN for TSS; (I–L) PLS, SVR, CUB, and QRNN for *E. coli*. The blue and yellow data points represent the training and testing datasets, respectively. The black dashed lines represent the line of equality (*y* = *x*). The comparative evaluation among these models showed that the SVR had the best prediction performance for COD and *E. coli*; the CUB had the best prediction for TSS.

Among the models, SVR showed the best performance for COD prediction with the lowest RMSE of 270 mg/L, indicating its superior predictive accuracy over the other models (Figure 5A–D). Furthermore, the *R*^2^ of the testing dataset was 0.96, indicating that the model was likely not overfitted. This is consistent with the *R*^2^ value obtained for the training dataset, which was 0.99 (as shown in Table S2), and the slight degradation observed is within the expected range. The CUB model also demonstrated acceptable prediction accuracy, with a RMSE of 285 mg/L and *R*^2^ of 0.94. For COD prediction, the SVR model also had the best MAPE with 14.5%. Although the best MAPE exceeded 10%, it was still acceptable given that the COD concentrations spanned from 0.5 to 5820 mg/L.

The top-performing model for TSS prediction was CUB, achieving a test RMSE of 55 mg/L and *R*^2^ of 0.99 (Figure 5E–H). In contrast, the PLS linear model had a poor prediction on TSS, particularly for concentrations lower than 100 mg/L. Meanwhile, the QRNN had poor predictions for high concentrations (>500 mg/L) of TSS. The SVR and CUB models both had a low MAPE of 24.1 and 24.8%, respectively. The SVR model had a higher RMSE than the CUB model but a lower MAPE, which could be attributed to its larger error when predicting higher TSS concentrations.

Although the SVR model had the lowest RMSE of 7.38 × 10^6^ MPN/100 mL and *R*^2^ of 0.83, which was the best performing model for *E. coli* prediction, it can be seen from the high RMSE that all models had inaccuracies when predicting *E. coli* (Figure 5I–L). This low prediction accuracy is likely due to the significant concentration differences between the sampling points (Table S1). After membrane filtration and chlorination, the *E. coli* concentrations were often BDL, which caused the Perm, P-NCS, and effluent samples to lose variation in their concentrations. As variations are essential to train predictive models, this resulted in high *E. coli* concentrations dominating the dataset, which skewed the regression models. This is particularly true for PLS, where most predictions fell in the 10^5^ to 10^7^ MPN/100 mL range. QRNN also had predictions that were around 10^4^ MPN/100 mL, while SVM and CUB could predict lower concentrations of *E. coli*, although their accuracy was not ideal. Overall, the SVR model was the best at predicting COD, and the CUB model was the best at predicting TSS. The accuracy in *E. coli* prediction could be improved by applying methods such as a classification, log-transformation, or joint discrete-continuous model that consider a wider range of concentration differences during model development, such as those observed across the multiple sampling points used in this study.

### 3.4 Additional Observations from the Model Prediction

Apart from the comparative evaluation of the models, the spatial prediction performance was also assessed across the five sampling points (Table 2 and Figures S4–S6). According to the findings presented in Table 2, the SVR exhibited superior prediction performance across most sampling points when predicting COD, surpassing the performance of the other models. However, it was evident that the SVR model had limitations in predicting the COD concentration for Inf samples. The CUB shows the lowest RMSE and highest *R*^2^, indicating that the decision tree/linear-based CUB had better predictions for high COD concentrations. The prediction accuracy of all models decreased for all post-membrane stages (Perm, P-NCS, and Eff) compared to the COD predictions of the AnMBR. However, considering that the system’s average P-NCS and Eff COD concentration was 117.74±90.80 and 127.48 ±87.34 mg/L, respectively, the SVR had the most accurate predictions of all models, with a RMSE of 31 and 37 mg/L for P-NCS and Eff.

**Table 2:** Comparative Evaluation of Testing Dataset RMSE and *R*^2^ of COD, TSS, and *E. coli* in Each Sampling Point

sampling point	model structure	COD (mg/L)		TSS (mg/L)		*E. coli* (MPN/100 mL)	
		RMSE	*R*^2^	RMSE	*R*^2^	RMSE	*R*^2^
influent	PLS	536	0.52	214	0.30	9.05 × 10^6^	0.32
	SVR	522	0.29	152	0.69	1.04 × 10^7^	0.01
	CUB	322	0.82	48	0.97	8.86 × 10^6^	0.02
	QRNN	452	0.45	208	0.51	6.30 × 10^6^	0.16
AnMBR	PLS	503	0.85	518	0.39	1.23 × 10^7^	0.01
	SVR	177	0.98	154	0.91	9.81 × 10^6^	0.01
	CUB	595	0.80	100	0.96	8.60 × 10^6^	0.12
	QRNN	294	0.94	602	0.34	1.19 × 10^7^	0.02
permeate	PLS	245	0.26	122	0.25	3.80 × 10^6^	0.03
	SVR	93	0.75	2.2	0.57	2.93 × 10^5^	0.29
	CUB	107	0.11	3.2	0.69	6.44 × 10^3^	0.28
	QRNN	241	0.16	12	0.32	5.53 × 10^3^	0.13
post-NCS	PLS	150	0.68	37	0.20	1.21 × 10^6^	<0.01
	SVR	31	0.85	1.5	0.68	6.46 × 10^3^	<0.01
	CUB	63	0.65	3.3	0.46	1.16 × 10^3^	<0.01
	QRNN	56	0.67	18	0.22	4.57 × 10^3^	<0.01
effluent	PLS	127	0.37	69	0.09	1.81 × 10^6^	<0.01
	SVR	37	0.94	4.4	0.75	1.85 × 10^3^	<0.01
	CUB	55	0.88	4.24	0.81	1.19 × 10^3^	<0.01
	QRNN	56	0.88	14	0.20	5.63 × 10^3^	<0.01

Compared to the other models, the CUB showed excellent prediction performance for Inf TSS concentration, with a RMSE of 48 mg/L. The CUB was stronger at identifying non-linear relationships by decision tree-based modeling and achieved more accurate predictions.^47^ When modeling the post-membrane treatment steps, the CUB maintained some accuracy; however, the RMSE of the SVR model was lower than the CUB model.

As mentioned previously, the prediction performance of *E. coli* was poor, especially when concentrations were BDL, as was the case in Perm, P-NCS, and Eff. The *R*^2^ was less than 0.01, indicating that the model’s accuracy was extremely low. Replacing the BDL *E. coli* data with 1 MPN/100 mL, or any discrete value between 0 and 1 MPN/100 mL, was not an ideal solution for improving the regression predictions. The measured *E. coli* levels spanned several orders of magnitude, ranging from 1 to 1.54 × 10^8^ MPN/100 mL. Although appropriate methods exist for processing data BDL, such as censored regression and dropping observations, which can obtain unbiased estimators of the model parameters, these methods were not applicable to this study as the missing BDL data were not random. However, it does indicate that ML classification can be useful in predicting whether pathogens in the effluent exceed a regulatory threshold. This approach can be a relevant strategy for monitoring OWTS operation.^21^ Further discussion on using classification ML algorithms to predict *E. coli* can be found in Supplementary Information (Section A.6), where the results indicate that the classification model achieves far superior prediction performance than the regression model.

## 4 Discussion and Limitations

This study aimed to develop soft sensor models for monitoring the off-line water parameters of COD, TSS, and *E. coli* in OWTSs. The models were trained and evaluated with 2 years of field test data from an existing OWTS. The results showed that the SVR had the best prediction accuracy for COD, while the CUB was the most effective for predicting TSS concentrations. However, due to *E. coli* levels spanning several orders of magnitude and the majority of algorithms being skewed by high values, the prediction results for *E. coli* were unsatisfactory and require further improvement. The best soft sensor models for predicting COD and TSS values were determined as having a MAPE of below 25%, which was considered acceptable performance for water quality monitoring of an OWTS. Furthermore, it was discovered that the different ML algorithms had variable predictive performance based on the sampling point and concentration levels due to changes in the chemical composition of the water samples. For instance, the CUB model demonstrated excellent accuracy in predicting high concentrations of COD and TSS. This can be attributed to the treatment effectiveness of the wastewater samples as they underwent the various treatment processes across the NG system.

This study highlights the potential of using in-line sensors and datasets of limited size to develop a soft sensor for monitoring the treatment performance of an OWTS by predicting critical off-line water quality parameters. The failure of OWTSs can often be attributed to their lack of a strict management plan and performance monitoring. OWTS operation and maintenance are usually the responsibility of homeowners or, in rare cases, local government/non-governmental organizations. These entities are often overburdened with other requirements, so lapses in maintenance and monitoring are common.^48^ Utilizing ML tools to develop a soft sensor for OWTSs can provide critical performance data to operators or stakeholders in remote and challenging areas. This would allow operators to know when the OWTS needs to be serviced and would avoid negative environmental effects caused by system failures.

However, the unavoidable limitations of ML predictions are that they tend to be site-specific. Changes in the physical and chemical characteristics of water quality at different sites can cause discrepancies in the soft sensor’s predictions and would require model calibrations between sites and applications. Although the soft sensor developed in this study achieved acceptable prediction results with only 56 data points, increasing the amount of raw data would reduce noise and uncertainty leading to more robust models. While the post-restart event data were omitted in this study, they may be incorporated in future soft sensors to indicate non-steady state operation and when system maintenance is needed. It is worth noting that the NH_4_^+^ and NO_3_^–^ measurements used in this study were obtained through standard colorimetry rather than ion-selective electrodes. If ion-selective electrodes were used, the model’s accuracy might be impacted due to sensor drift and ion interference. It should also be noted that the soft sensors developed by this study relied on properly maintained and calibrated sensors and analytical methods. However, as the resources to maintain in-line sensors will not always be available for OWTS systems, a long-term study is required to investigate how sensor maintenance frequency can impact predictive performance of the models. Schneider et al.^49^ compared the accuracy of three maintained and unmaintained in-line sensors, including pH, DO, and ORP, and indicated that the soft sensor based on the unmaintained sensors could still achieve a final prediction accuracy of over 90%. Lastly, the predictive performance of the model can be improved by adding additional variables, such as the system’s hydraulic loading rate (HRT), flow rate, ORP, DO, free chlorine concentrations, UV254, and so on. HRT and flow are important parameters when designing the degradation rate of substrates in a bioreactor; a lower HRT or higher flow rate usually results in insufficient contact time between the substrate and microbes, which lowers treatment efficiency.^50^ There are relatively stable ORP and DO sensors, which are used in WWTPs to control aeration and nitrification processes.^51^ For free chlorine concentrations and UV254, these two parameters are often used to monitor treatment processes when detecting the chlorination residual and organic matter in drinking water. Increasing the number of in-line sensors as well as the sampling datasets can improve the soft sensor prediction accuracy to a certain degree. However, further model applications to other OWTS are needed to assess the site specificity of the results. Overall, the soft sensors developed with ML algorithms and the real field test data in the study are the steppingstone for future development for OWTS monitoring. These ML-based soft sensors provide real-time monitoring that can be used to make adjustments to OWTS operations in remote areas for effective onsite treatment of wastewater.

## Supporting Information Available

Physical/chemical characteristics of sampled wastewater during the field trial of NG, graphical result of recursive feature elimination analysis (RFE), additional algorithms description on the comparative evaluation of the linear regression (LM), k-nearest neighbor (KNN), and random forest (RF), graphical result of feature importance analysis, and graphical result of standardized residual analysis (PDF)

## References

[ref1] Schaider L. A.; Rodgers K. M.; Rudel R. A. Review of Organic Wastewater Compound Concentrations and Removal in Onsite Wastewater Treatment Systems. Environ. Sci. Technol. 2017, 51, 7304–7317. 10.1021/acs.est.6b04778. 28617596

[ref2] Cid C. A.; Abiola F.; Starkl M. Can International Nonsewered Sanitation Standards Help Solve the Global Sanitation Crisis?. Environ. Sci. Technol. 2022, 56, 699–706. 10.1021/acs.est.1c03471. 34982549

[ref3] Borchardt M. A.; Bradbury K. R.; Alexander E. C. Jr.; Kolberg R. J.; Alexander S. C.; Archer J. R.; Braatz L. A.; Forest B. M.; Green J. A.; Spencer S. K. Norovirus Outbreak Caused by a New Septic System in a Dolomite Aquifer. Groundwater 2011, 49, 85–97. 10.1111/j.1745-6584.2010.00686.x. 20199588

[ref4] Marshall R.; Levison J.; Parker B.; McBean E. Septic System Impacts on Source Water: Two Novel Field Tracer Experiments in Fractured Sedimentary Bedrock. Sustainability 2022, 14, 1959 10.3390/su14041959.

[ref5] Halicki W.; Halicki M. Effective Removal of Biogenic Substances Using Natural Treatment Systems for Wastewater for Safer Water Reuse. Water 2022, 14, 3977 10.3390/w14233977.

[ref6] Korostynska O.; Mason A.; Al-Shamma’a A. I. Monitoring Pollutants in Wastewater: Traditional Lab Based versus Modern Real-Time Approaches. In Smart Sensors for Real-Time Water Quality Monitoring; Mukhopadhyay S. C., Mason A., Eds.; Smart Sensors, Measurement and Instrumentation; Springer: Berlin, Heidelberg, 2013; pp 1–24.

[ref7] Zhang W.; Tooker B.; Mueller V. Enabling Wastewater Treatment Process Automation: Leveraging Innovations in Real-Time Sensing, Data Analysis, and Online Controls. Environ. Sci.: Water Res. Technol. 2020, 6, 2973–2992. 10.1039/D0EW00394H.

[ref8] Corominas L.; Garrido-Baserba M.; Villez K.; Olsson G.; Cortés U.; Poch M. Transforming Data into Knowledge for Improved Wastewater Treatment Operation: A Critical Review of Techniques. Environ. Model. Software 2018, 106, 89–103. 10.1016/j.envsoft.2017.11.023.

[ref9] The Water Environment Federation. Automation of Wastewater Treatment Facilities - WEF MoP 21, Third Edition, 3rd ed.; McGraw-Hill Education: New York, 2007; pp 174–181.

[ref10] Ge L.; Wang P.; Ge S.; Li N.; Yu J.; Yan M.; Huang J. Photoelectrochemical Lab-on-Paper Device Based on an Integrated Paper Supercapacitor and Internal Light Source. Anal. Chem. 2013, 85, 3961–3970. 10.1021/ac4001496. 23472854

[ref11] Chang P.; Li Z. Over-Complete Deep Recurrent Neutral Network Based on Wastewater Treatment Process Soft Sensor Application. Appl. Soft Comput. 2021, 105, 107227 10.1016/j.asoc.2021.107227.

[ref12] Spectrometer Probes. s::can. https://www.s-can.at/en/our-products/spectrometer-probes/ (accessed May 20, 2022).

[ref13] EZ Series COD Analyzers. Hach. https://www.hach.com/p-ez-series-cod-analyzers/EZ7004.XXXXXXXX (accessed May 20, 2022).

[ref14] IQ SensorNet Optical UV Absorption Probe. ysi. https://www.ysi.com/uvt-254 (accessed May 20, 2022).

[ref15] Huang M.; Ma Y.; Wan J.; Chen X. A Sensor-Software Based on a Genetic Algorithm-Based Neural Fuzzy System for Modeling and Simulating a Wastewater Treatment Process. Appl. Soft Comput. 2015, 27, 1–10. 10.1016/j.asoc.2014.10.034.

[ref100] Lambrou T. P.; Anastasiou C. C.; Panayiotou C. G.; Polycarpou M. M. A Low-Cost Sensor Network for Real-Time Monitoring and Contamination Detection in Drinking Water Distribution Systems. IEEE Sensors J. 2014, 14 (8), 2765–2772. 10.1109/JSEN.2014.2316414.

[ref16] Pattnaik B. S.; Pattanayak A. S.; Udgata S. K.; Panda A. K. Machine Learning Based Soft Sensor Model for BOD Estimation Using Intelligence at Edge. Complex Intell. Syst. 2021, 7, 961–976. 10.1007/s40747-020-00259-9.

[ref17] Zhu M.; Wang J.; Yang X.; Zhang Y.; Zhang L.; Ren H.; Wu B.; Ye L. A Review of the Application of Machine Learning in Water Quality Evaluation. Eco-Environ. Health 2022, 1, 107–116. 10.1016/j.eehl.2022.06.001. PMC10702893 38075524

[ref18] Paepae T.; Bokoro P. N.; Kyamakya K. From Fully Physical to Virtual Sensing for Water Quality Assessment: A Comprehensive Review of the Relevant State-of-the-Art. Sensors 2021, 21, 6971 10.3390/s21216971. 34770278 PMC8587795

[ref19] Sivchenko N.; Kvaal K.; Ratnaweera H. Floc Sensor Prototype Tested in the Municipal Wastewater Treatment Plant. Cogent Eng. 2018, 5, 1436929 10.1080/23311916.2018.1436929.

[ref20] Foschi J.; Turolla A.; Antonelli M. Soft Sensor Predictor of *E. coli* Concentration Based on Conventional Monitoring Parameters for Wastewater Disinfection Control. Water Res. 2021, 191, 116806 10.1016/j.watres.2021.116806. 33454652

[ref21] Qin X.; Gao F.; Chen G. Wastewater Quality Monitoring System Using Sensor Fusion and Machine Learning Techniques. Water Res. 2012, 46, 1133–1144. 10.1016/j.watres.2011.12.005. 22200261

[ref22] Yang Y.; Kim K.-R.; Kou R.; Li Y.; Fu J.; Zhao L.; Liu H. Prediction of Effluent Quality in a Wastewater Treatment Plant by Dynamic Neural Network Modeling. Process Saf. Environ. Prot. 2022, 158, 515–524. 10.1016/j.psep.2021.12.034.

[ref23] Monitoring And Reporting Program For Regional Board Order No. R4–2004-0146 General Waste Discharge Requirements for Residential Onsite Wastewater Treatment Systems; California State Water Resources Control Board. https://www.waterboards.ca.gov/losangeles/board_decisions/adopted_orders/general_orders/r4-2004-0146/r4-2004-0146_att_c.pdf (accessed April 28, 2022).

[ref24] Onsite Wastewater Treatment Survey and Assessment. State of Hawaii Department of Business, Economic Development and Tourism Office of Planning, Hawaii Coastal Zone Management Program Department of Health. https://health.hawaii.gov/wastewater/files/2013/06/onsitesurvey.pdf (accessed April 28, 2022).

[ref25] Performance requirements; laboratory sampling and monitoring. Virginia Law. https://law.lis.virginia.gov/admincode/title12/agency5/chapter613/section100/ (accessed April 28, 2022).

[ref26] Ching P. M. L.; So R. H. Y.; Morck T. Advances in Soft Sensors for Wastewater Treatment Plants: A Systematic Review. J. Water Process Eng. 2021, 44, 102367 10.1016/j.jwpe.2021.102367.

[ref27] Souza F. A. A.; Araújo R.; Mendes J. Review of Soft Sensor Methods for Regression Applications. Chemom. Intell. Lab. Syst. 2016, 152, 69–79. 10.1016/j.chemolab.2015.12.011.

[ref28] Shyu H.-Y.; Bair R. A.; Castro C. J.; Xaba L.; Delgado-Navarro M.; Sindall R.; Cottingham R.; Uman A. E.; Buckley C. A.; Yeh D. H. The NEWgeneratorTM Non-Sewered Sanitation System: Long-Term Field Testing at an Informal Settlement Community in EThekwini Municipality, South Africa. J. Environ. Manage. 2021, 296, 112921 10.1016/j.jenvman.2021.112921. 34303262 PMC8404038

[ref29] Castro C. J.; Shyu H. Y.; Xaba L.; Bair R.; Yeh D. H. Performance and Onsite Regeneration of Natural Zeolite for Ammonium Removal in a Field-Scale Non-Sewered Sanitation System. Sci. Total Environ. 2021, 776, 145938 10.1016/j.scitotenv.2021.145938. 33652315 PMC8111385

[ref30] Rice E. W.; Bridgewater L.; Association, A. P. H. Standard Methods for the Examination of Water and Wastewater; American public health association: Washington, DC, 2012; Vol. 10.

[ref31] Walpole R. E.; Myers R. H.; Myers S. L.; Ye K. Probability and Statistics for Engineers and Scientists; Macmillan: New York, 1993; Vol. 5, pp 326 −332.

[ref32] Singh N. K.; Yadav M.; Singh V.; Padhiyar H.; Kumar V.; Bhatia S. K.; Show P.-L. Artificial Intelligence and Machine Learning-Based Monitoring and Design of Biological Wastewater Treatment Systems. Bioresour. Technol. 2023, 369, 128486 10.1016/j.biortech.2022.128486. 36528177

[ref33] Cannon A. J. Quantile Regression Neural Networks: Implementation in R and Application to Precipitation Downscaling. Comput. Geosci. 2011, 37, 1277–1284. 10.1016/j.cageo.2010.07.005.

[ref34] Wang L.; Long F.; Liao W.; Liu H. Prediction of Anaerobic Digestion Performance and Identification of Critical Operational Parameters Using Machine Learning Algorithms. Bioresour. Technol. 2020, 298, 122495 10.1016/j.biortech.2019.122495. 31830658

[ref35] R Core Team. R: A Language and Environment for Statistical Computing, 2013. http://www.R-project.org/ (accessed April 28, 2022).

[ref36] Kuhn M. Building Predictive Models in R Using the Caret Package. J. Stat. Software 2008, 28, 1–26. 10.18637/jss.v028.i05.

[ref37] Geladi P.; Kowalski B. R. Partial Least-Squares Regression: A Tutorial. Anal. Chim. Acta 1986, 185, 1–17. 10.1016/0003-2670(86)80028-9.

[ref38] Burges C. J. C. A Tutorial on Support Vector Machines for Pattern Recognition. Data Min. Knowl. Discovery 1998, 2, 121–167. 10.1023/A:1009715923555.

[ref39] Quinlan J. R. Combining Instance-Based and Model-Based Learning. In Proceedings of the tenth international conference on machine learning; 1993; pp 236–243. (1)

[ref40] Bagherzadeh F.; Mehrani M.-J.; Basirifard M.; Roostaei J. Comparative Study on Total Nitrogen Prediction in Wastewater Treatment Plant and Effect of Various Feature Selection Methods on Machine Learning Algorithms Performance. J. Water Process Eng. 2021, 41, 102033 10.1016/j.jwpe.2021.102033.

[ref41] Kuhn M.; Johnson K. Applied Predictive Modeling; Springer, 2013; Vol. 26; pp 500–502.

[ref42] Kumar A.; Samadder S. R.; Kumar N.; Singh C. Estimation of the Generation Rate of Different Types of Plastic Wastes and Possible Revenue Recovery from Informal Recycling. Waste Manage. 2018, 79, 781–790. 10.1016/j.wasman.2018.08.045. 30343811

[ref43] Guo H.; Jeong K.; Lim J.; Jo J.; Kim Y. M.; Park J.; Kim J. H.; Cho K. H. Prediction of Effluent Concentration in a Wastewater Treatment Plant Using Machine Learning Models. J. Environ. Sci. 2015, 32, 90–101. 10.1016/j.jes.2015.01.007. 26040735

[ref44] Kadlec P.; Gabrys B.; Strandt S. Data-Driven Soft Sensors in the Process Industry. Comput. Chem. Eng. 2009, 33, 795–814. 10.1016/j.compchemeng.2008.12.012.

[ref45] Eddy M.; Abu-Orf M.; Bowden G.; Burton F. L.; Pfrang W.; Stensel H. D.; Tchobanoglous G.; Tsuchihashi R.; AECOM (Firm). Wastewater Engineering: Treatment and Resource Recovery; McGraw Hill Education, 2014; pp 354–388.

[ref46] Abba S. I.; Pham Q. B.; Usman A. G.; Linh N. T. T.; Aliyu D. S.; Nguyen Q.; Bach Q.-V. Emerging Evolutionary Algorithm Integrated with Kernel Principal Component Analysis for Modeling the Performance of a Water Treatment Plant. J. Water Process Eng. 2020, 33, 101081 10.1016/j.jwpe.2019.101081.

[ref47] Noi P. T.; Degener J.; Kappas M. Comparison of Multiple Linear Regression, Cubist Regression, and Random Forest Algorithms to Estimate Daily Air Surface Temperature from Dynamic Combinations of MODIS LST Data. Remote Sens. 2017, 9, 398 10.3390/rs9050398.

[ref48] Massoud M. A.; Tarhini A.; Nasr J. A. Decentralized Approaches to Wastewater Treatment and Management: Applicability in Developing Countries. J. Environ. Manage. 2009, 90, 652–659. 10.1016/j.jenvman.2008.07.001. 18701206

[ref49] Schneider M. Y.; Carbajal J. P.; Furrer V.; Sterkele B.; Maurer M.; Villez K. Beyond Signal Quality: The Value of Unmaintained PH, Dissolved Oxygen, and Oxidation-Reduction Potential Sensors for Remote Performance Monitoring of on-Site Sequencing Batch Reactors. Water Res. 2019, 161, 639–651. 10.1016/j.watres.2019.06.007. 31254889

[ref50] Rittmann B. E.; McCarty P. L. Environmental Biotechnology: Principles and Applications; McGraw-Hill Education, 2001; pp 645–666.

[ref51] Olsson G.; Carlsson B.; Comas J.; Copp J.; Gernaey K. V.; Ingildsen P.; Jeppsson U.; Kim C.; Rieger L.; Rodríguez-Roda I.; Steyer J.-P.; Takács I.; Vanrolleghem P. A.; Vargas A.; Yuan Z.; Åmand L. Instrumentation, Control and Automation in Wastewater – from London 1973 to Narbonne 2013. Water Sci. Technol. 2014, 69, 1373–1385. 10.2166/wst.2014.057. 24718326

