# Supplementary material for: Development of a Soft Sensor Using Machine Learning Algorithms for Predicting the Water Quality of an Onsite Wastewater Treatment System
Source: ACS Environ Au. 2023 Jun 30;3(5):308–18. doi: 10.1021/acsenvironau.2c00072 (PMC10515708; doi:10.1021/acsenvironau.2c00072)
Supplement: Supplementary file 1 — vg2c00072_si_002.pdf [file vg2c00072_si_002.pdf]

A. Supplementary Information: Development of a soft sensor using machine learning algorithms for predicting the water quality of an onsite wastewater treatment system

Hsiang-Yang Shyu [†], Cynthia J. Castro [†], Robert A. Bair [†], Qing Lu [†], Daniel H. Yeh[†, *]

[†] University of South Florida, Civil & Environmental Engineering, 4202 E. Fowler Ave., Tampa, FL 33620, United States

*Corresponding Author (dhyeh@usf.edu)

A.1. Comparison of k-fold cross-validation with RMSE

This section compares root mean square error (RMSE) values across different values of k to determine the optimal k for a k-fold cross-validation when training the model. K-fold cross-validation is a widely used technique for model selection and performance evaluation in ML algorithms. It involves dividing the dataset into k subsets of equal size, with k-1 subsets used for training the model and one subset for testing it. This process is repeated k times, with each subset used once for testing, while the final performance metric is calculated as the average of the RMSE values obtained from the k-testing sets. The "repeatedcv" function with k=3, 5, 10, 15, and 20, and 3 repeats, as implemented in the R caret package, was utilized in this study[1]. This specific cross-validation method aims to provide a robust estimation of model performance by repeating the k-fold cross-validation process multiple times. It helps to reduce the impact of randomness in the partitioning of data into folds and ensures the validity and generalizability of the results.

Figure S1 shows that increasing k leads to lower RMSE. This signifies that the dataset will have better model performance with increased k values. Larger k values allow each fold to contain a smaller portion of the dataset, resulting in more data used for training. However, the reduction in RMSE decreases at higher k values, indicating diminishing value of using higher k values. For predicting COD, the average RMSE decreased by 3.26%, 5.53%, 2.25%, and 1.25% between k=3, 5, 10, 15, and 20, respectively. The average RMSE in predicting TSS decreased by 0.51%, 11.89%, 7.91%, and 5.74%, while the average RMSE in predicting *E. coli* decreased by 17.25%, 11.70%, 6.43%, and 4.39%, respectively. Considering the cost of increased computational time, which

increases by 1.5 times between 15 to 20-fold, and the slowdown in the downward trend of RMSE with k, a 15-fold cross-validation was chosen for this study.

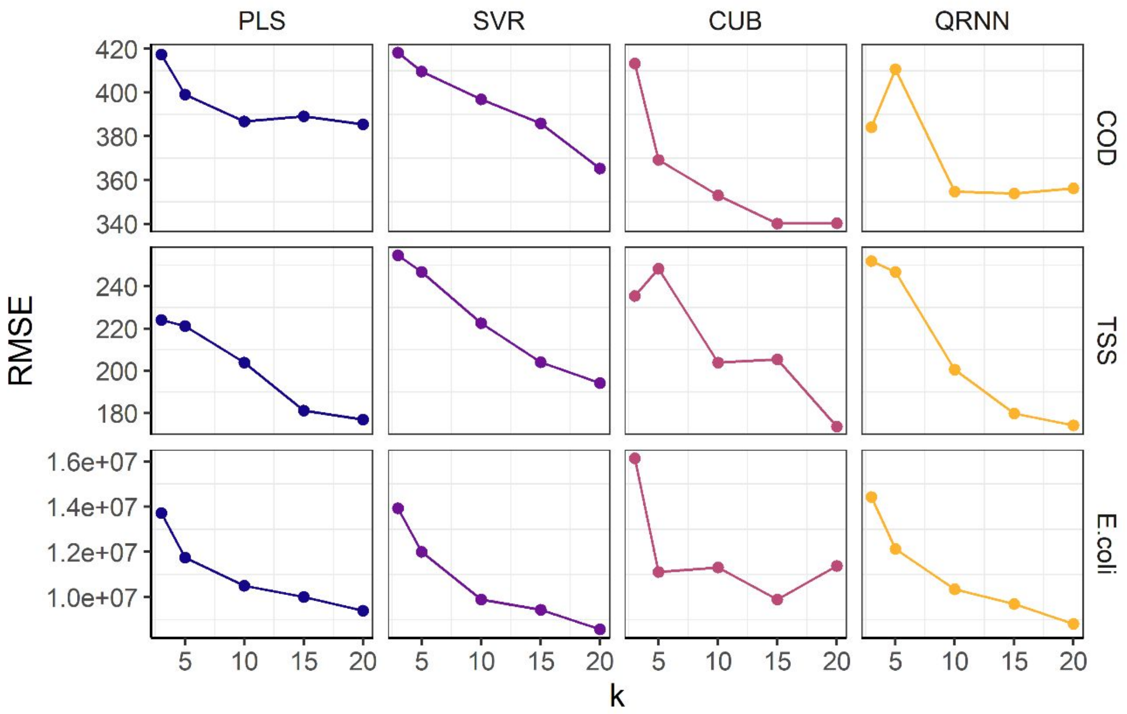

Figure S1: Comparison of k-fold cross-validation with RMSE was performed for three output variables (COD, TSS, and E. coli) using four ML algorithms (PLS, SVR, CUB, and QRNN) trained using the training dataset.

A.2. Z-score analysis

This section utilizes the z-score analysis to identify the non-steady state performance of the system and eliminate outliers from the dataset. Three significant restart events occurred during the field trial, resulting in abnormal spikes in various water quality parameters. These events include the December 2018 summer vacation shutdown, the August 2019 nutrient capture system (NCS) maintenance, and the December 2019 shutdown with NCS maintenance in January 2020. The daily z-score was calculated by averaging the z-score values of individual water quality parameters, including COD, TSS, *E. coli*, turbidity, color, pH, NH_4^+, NO_3^-, and electrical conductivity (EC). A daily z-score value greater than two times the standard deviation was considered non-steady state performance. The results of the z-score analysis, presented in Figure S2, indicate that the NCS maintenance events significantly impacted the water quality parameters. Additionally, Figure S2 shows that the abnormal spikes in the z-score caused by the NCS maintenance events returned to steady state operations after two weeks. As a result, two weeks of data following system disruptions and maintenance events were eliminated from the training set to reduce noise.

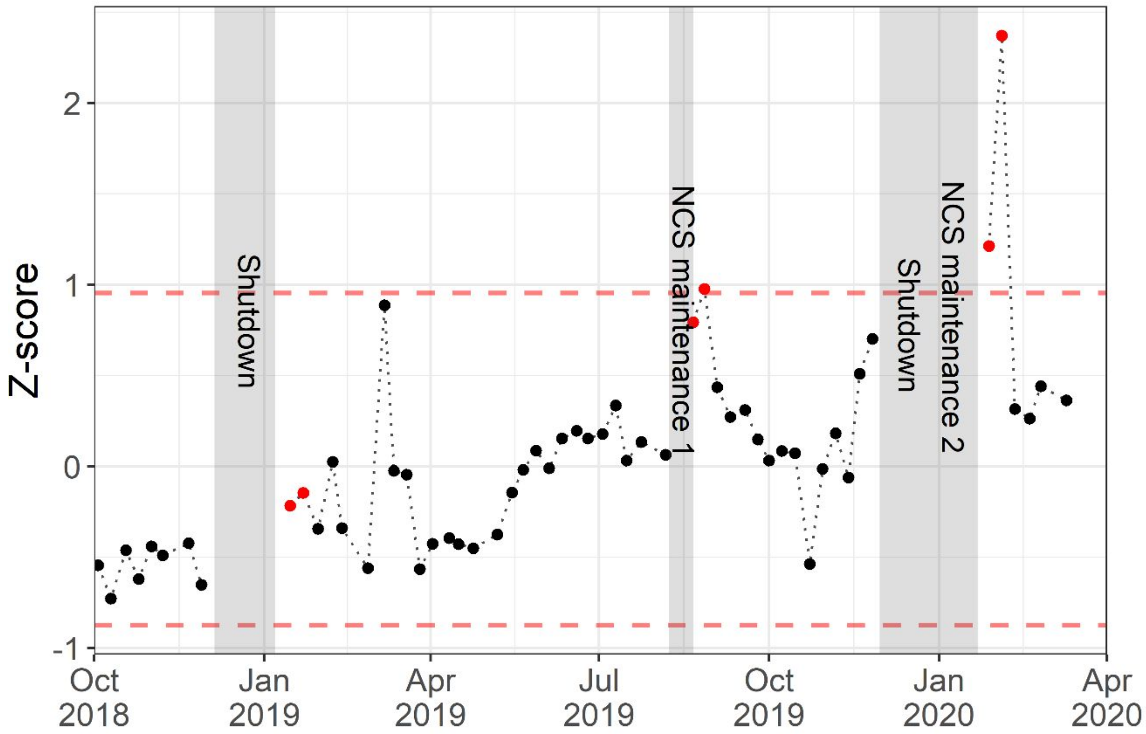

Figure S2: Z-score analysis for the water quality. The dots represent the daily mean z-score of the water quality parameters, while the red dots indicate the data points occurring after restart or maintenance events. The red dashed line represents the two standard deviations from the mean.

A.3. Characteristics of Sampled Wastewater

Table S1: Physical/chemical characteristics of sampled wastewater during the field for five sampling points in NG (n =56).

Sampling point	Parameters	Mean	Standard deviation	Min	Max	Range
Influent	COD (mg/L)	2,420	967.25	1,051	5,820	4,769
	SCOD (mg/L)	1,310	530.75	460.00	2,885	2,425
	TSS (mg/L)	653	391.85	38	1,975	1,937
	E. coli $(MPN/100mL)$	9.96×10^{6}	2.49×10^{7}	2.41×10^{3}	1.54×10^{8}	1.53×10^{8}
	Color (Pt/Co)	1,908	1,104.95	45	4,955	4,910
	Turbidity (NTU)	967	611.95	402	4,480	4,078
	Conductivity (µs/cm)	2,404	847.65	502	3,880	3,378
	pH	6.75	0.52	5.57	8.90	3.34
	NH_4^+ (mg/L)	218	100.60	11	480	469
	NO_3^- (mg/L)	2	1.14	0.5	5.7	5.2
	Temperature (°C)	23.28	2.49	18.94	28.24	9.30
AnMBR	COD (mg/L)	3,244	1,897.96	533	7,855	7,322
	SCOD (mg/L)	1,949.57	1,528	322.50	7,015	6,693
	TSS (mg/L)	873	729.94	325	3,525	3,225
	E. coli $(MPN/100mL)$	4.94×10^{6}	9.24×10^{6}	1.84×10^{7}	4.84×10^{7}	3×10^{7}
	Color (Pt/Co)	3,944	6,970.78	98	49,600	49,502
	Turbidity (NTU)	1,731	1,130.37	315	4,385	4,069
	Conductivity (µs/cm)	2,793	863.75	1,273	4,735	3,462
	pH	6.98	0.53	6.09	9.68	3.58
	NH_4^+ (mg/L)	225	93.18	10.00	480	470
	NO_3^- (mg/L)	4	2.61	0.5	10.5	10
	Temperature (°C)	23.59	2.44	18.90	28.94	10.05
Permeate	COD (mg/L)	37.61	224.90	205	1,600	1,395
	SCOD (mg/L)	312.54	125.99	58	924	866
	TSS (mg/L)	9.68	23.32	1	147	146
	E. coli $(MPN/100mL)$	452	1,840.83	<2.2	12,098	12,098
	Color (Pt/Co)	778.88	764.56	10	3,580	3,570
	Turbidity (NTU)	188.04	105.14	6.89	579	572.11
	Conductivity (µs/cm)	2,769.57	866.50	1,170	4,810	3,640
	pH	7.07	0.66	6.09	11.07	4.99

	NH_4^+ (mg/L)	249.52	112.89	10	518	508
	NO_3^- (mg/L)	1.57	0.93	0.5	4.5	4
Post-NCS	COD (mg/L)	127.48	87.34	0.5	352.50	352
	SCOD (mg/L)	119.83	85.87	0.5	352.50	352
	TSS (mg/L)	12.08	19.12	0.5	120.50	120
	E. coli $(MPN/100mL)$	0.15	0.57	<2.2	3	3
	Color (Pt/Co)	246.12	381.42	0	2,500	2,500
	Turbidity (NTU)	50.49	101.95	0.41	708	707.59
	Conductivity (µs/cm)	2,476	1,053.17	689	5,065	4,376
	pH	7.49	0.90	6.11	11.36	5.25
	NH_4^+ (mg/L)	68.42	86.31	0.5	272.50	272
	NO_3^- (mg/L)	0.96	0.81	0.5	3.7	3.2
Effluent	COD (mg/L)	117.74	90.80	0.5	366.50	366
	SCOD (mg/L)	110.34	90.13	0.5	366.50	366
	TSS (mg/L)	10.25	10.17	0.5	56.67	56.17
	E. coli $(MPN/100mL)$	<2.2	0	<2.2	<2.2	0
	Color (Pt/Co)	155.62	266.58	0	1,750	1,750
	Turbidity (NTU)	35.77	92.44	0.50	686	685.50
	Conductivity (µs/cm)	2,531.83	1,115.74	457	5,330	4,873
	pH	7.54	0.78	6.20	11.45	5.25
	NH_4^+ (mg/L)	66.21	86.65	0.5	274	273.5
	NO_3^- (mg/L)	1.03	0.89	0.5	3.15	2.65

A.4. Result of Recursive Feature Elimination Analysis

To avoid bias when selecting the model input variables, the k-fold cross-validation was used in recursive feature elimination analysis (RFE). In RFE, the training dataset is divided into k = 15 subsets to reduce model overfitting when training. Figure S3 shows the RMSE and deviation results of the RFE analysis. The group with the lowest average RMSE was selected to represent the best selection of the variables for each model.

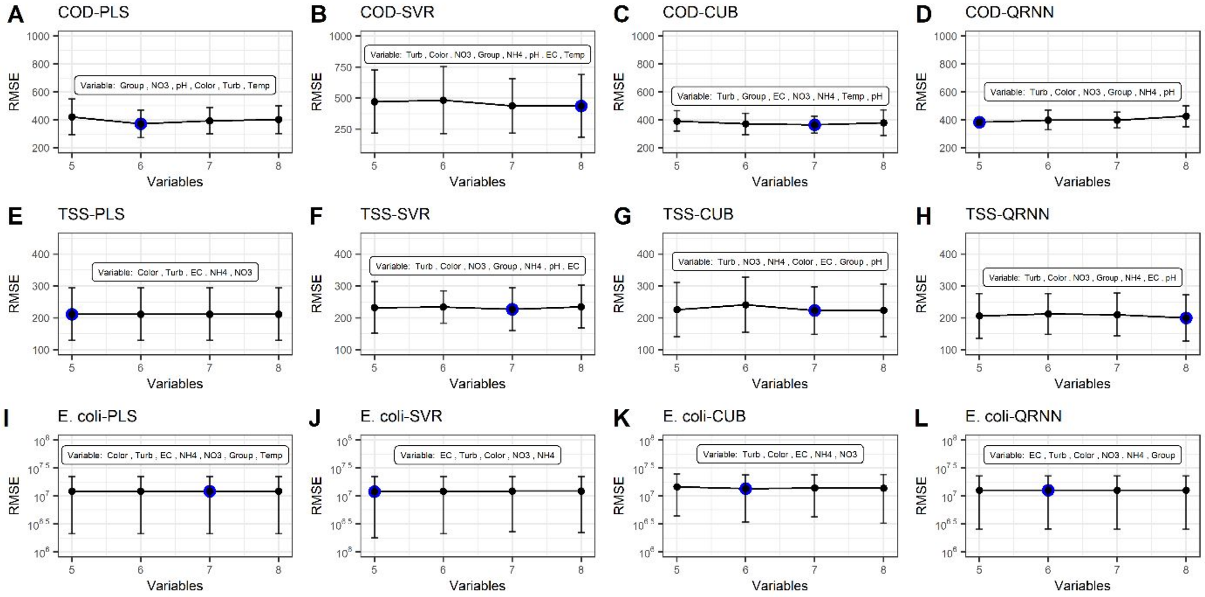

Figure S3: Recursive feature elimination analysis results on COD, TSS, and E. coli. (A-D) PLS, SVR, CUB, and QRNN for COD; (E-H) PLS, SVR, CUB, and QRNN for TSS; (I-L) PLS, SVR, CUB, and QRNN for E. coli. The blue point represents the lowest RMSE for the output variables in each model.

A.5. Additional Model Description

Table S2: RMSE, R^2, and MAPE computed on training and testing datasets for each machine learning model.

Variable (Unit)	Model structure	Training RMSE	Training R^2	Testing RMSE	Testing R^2	Testing MAPE (%)	Final hyperparameters
COD (mg/L)	PLS	331	0.93	358	0.93	31.3	ncomp = 5
	SVR	199	0.98	270	0.96	14.5	sigma = 0.01; cost = 5.5
	CUB	132	0.99	285	0.96	20.4	committees =4; neighbors = 3
	QRNN	332	0.93	284	0.96	24.3	n.hidden = 2; penalty = 31.6
TSS (mg/L)	PLS	168	0.86	289	0.67	56.7	ncomp = 3
	SVR	79	0.97	107	0.95	24.1	sigma = 0.05; cost = 17.5
	CUB	31	0.99	54	0.98	24.8	committees =9; neighbors = 3
	QRNN	156	0.88	321	0.61	46.9	n.hidden = 2; penalty = 57.2
E. coli ($MPN/100ml$	PLS	1.44×10^7	0.12	1.44×10^7	0.20	88.2	ncomp = 1
	SVR	7.38×10^6	0.91	7.38×10^7	0.83	83.5	sigma = 0.1; cost = 1.5
	CUB	7.96×10^6	0.60	7.96×10^7	0.22	71.4	committees =10; neighbors = 9
	QRNN	1.48×10^7	0.10	1.48×10^7	0.12	87.7	n.hidden = 4; penalty = 98.7

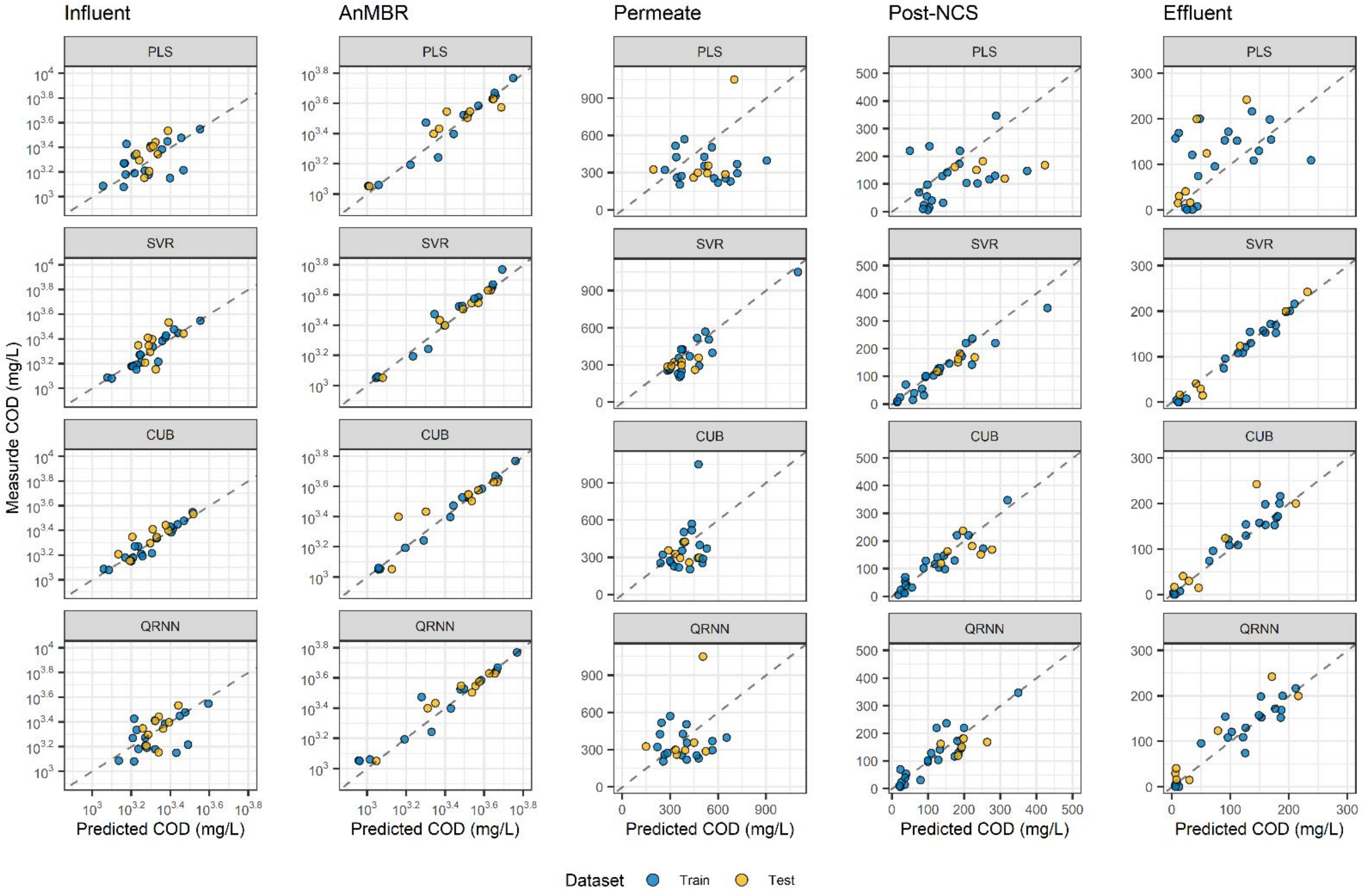

Figure S4: Model predictions for COD in each sampling point. The circle and triangle data points represent the testing and training datasets, respectively. The black dashed lines represent the line of equality (y = x). The comparative evaluation among these models showed that SVR had the best prediction performance for COD in the lower concentration sampling points (Permeate, Post-NCS, and Effluent), while CUB had better prediction performance in the higher COD concentration range sampling points (Influent and AnMBR).

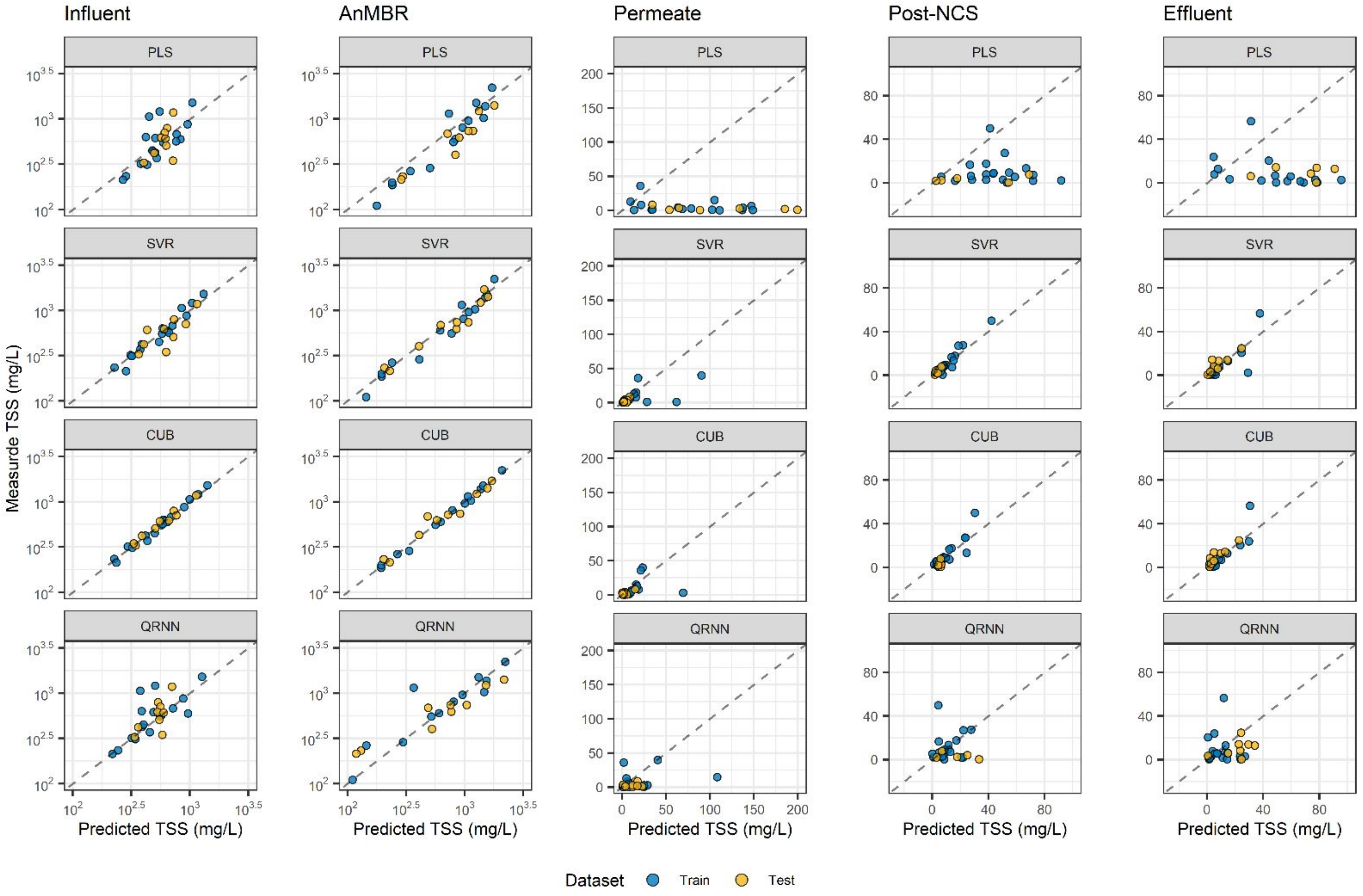

Figure S5: Model predictions for TSS in each sampling point. The circle and triangle data points represent the testing and training datasets, respectively. The black dashed lines represent the line of equality (y = x). The comparative evaluation among these models showed that CUB had the best prediction performance for TSS in the higher concentration range sampling points (Influent and AnMBR), while after the membrane process, CUB and SVR both showed well prediction accuracy.

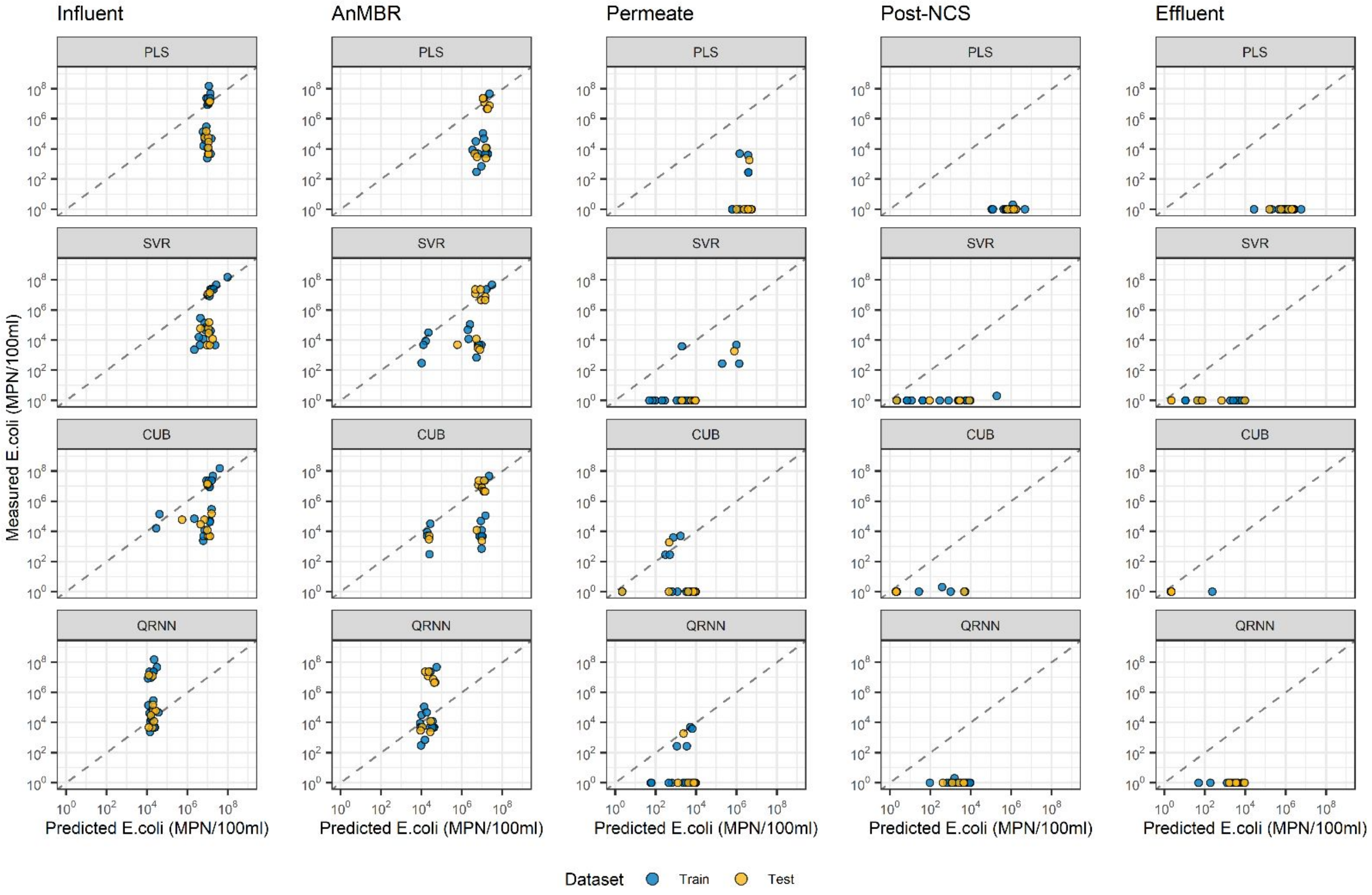

Figure S6: Model predictions for E. coli in each sampling point. The circle and triangle data points represent the testing and training datasets, respectively. The black dashed lines represent the line of equality (y = x).

A.6. *E. coli* Prediction Using Classification ML Algorithms

Given the poor results of the ML regression models in predicting *E. coli* concentrations, a classification method that was better suited to the *E. coli* data was investigated. The *E. coli* concentration data was broken down into four ranges: a high (H) concentration range for concentrations $> 10^7\ MPN/100mL$; a medium (M) concentration range for concentrations $\leq 10^7$ and $> 10^2\ MPN/100mL$; a low (L) concentration range for concentrations $\leq 10^2$ and $> 2.2\ MPN/100mL$; and a final concentration range for concentrations lower than the detection limit (LDL).

Except for SVR, which is suitable for classification data using the support vector machine (SVM), the other models used for regression prediction were not well suited to classification prediction. In this section, SVM was used to predict the concentration range of *E. coli*, while the prediction accuracy of SVM was compared with two other popular classification algorithms, k-nearest neighbor (KNN) and random forest (RF). KNN (package: 'knn') is a non-parametric classification method that uses observed data points and their weights to simulate the final predicted result. To prevent the model from overfitting noisy data, this study optimized the hyperparameter k from 3. RF (package: 'rf') is a nonlinear, supervised learning method that comprises multiple independent decision tree classifiers where the final predicted result is based on aggregating all decision trees' results. The hyperparameters for RF include the number of candidates considered at each decision tree (mtry) and the number of trees to grow in the set (ntree). To avoid overfitting the model to the training dataset, the same methodology of 15-fold cross-validation, and the dataset was split into 70:30 for training and testing.

To evaluate the performance of the predictive models, a range of metrics including balanced accuracy, precision, recall, and F1 score were evaluated. Precision indicated how many predicted positive cases are positive, while recall showed how the model correctly identified actual positives. The F1 score is the combined means of precision and recall, providing a single number that balances both metrics. Balanced accuracy considers the imbalance in the classes of the dataset and is calculated by averaging sensitivity and specificity, where sensitivity measures the proportion of actual positive cases that were correctly identified as positive by the model, while specificity measures the proportion of actual negative cases that were correctly identified as negative by the model.

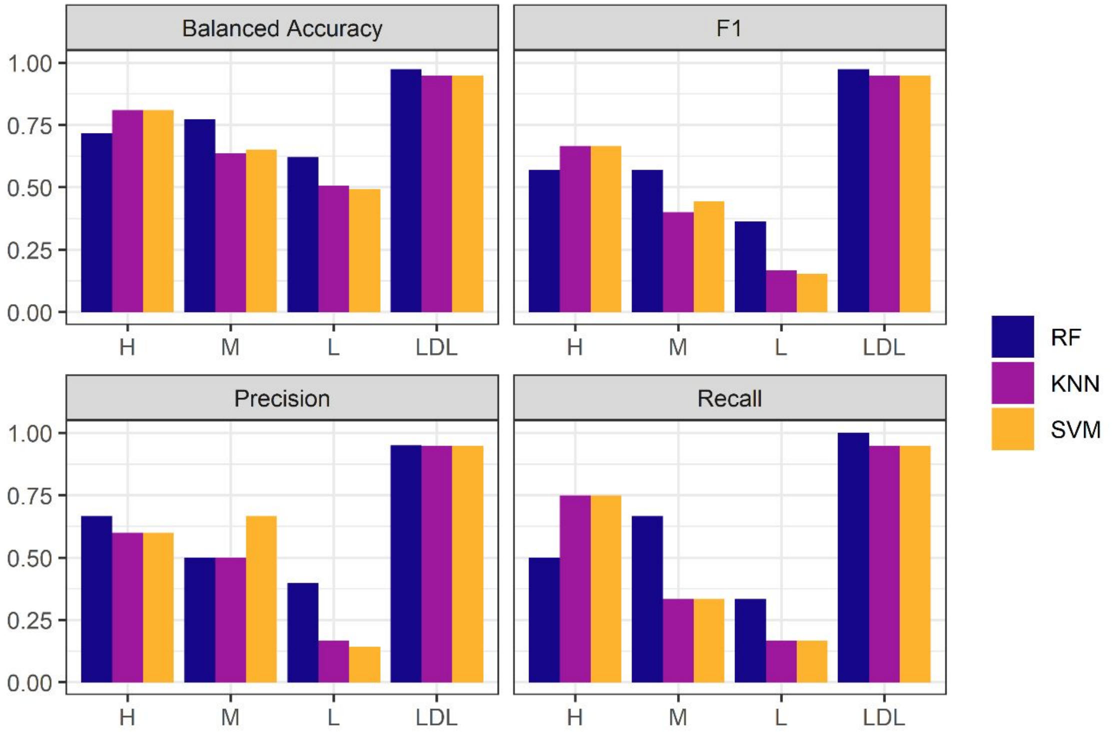

Figure S7: Comparison of performance metrics for three ML models. The bar chart shows the balanced accuracy, precision, recall, and F1 score for the RF, KNN, and SVM models.

The results demonstrate that RF had the highest predicted accuracy of 74.36%, followed by KNN and SVM with 69.23% each for the classification of *E. coli* concentration

ranges. Figure S7 illustrates that for the LDL range, all three models achieved a balanced accuracy of nearly 100%, while the L range had the lowest balanced accuracy of only around 50%, with SVM having the lowest accuracy of 49.24%. In the H and M range prediction, the balanced accuracy ranged from 65% to 80%, with KNN and SVM demonstrating slightly better predictions in the H range, while RF had slightly better predictions in the L range. Compared to the regression model, the classification model appeared to predict the presence of *E. coli* more effectively. Although it cannot provide exact values like the regression model, the classification model significantly improved the accuracy of predicting *E. coli*, thus making it feasible to use the constructed soft sensor to detect the concentration range of *E. coli*.

A.7. Reference

(1) Kuhn, M. Building Predictive Models in R Using the Caret Package. *J. Stat. Softw.* **2008**, *28*, 1–26. https://doi.org/10.18637/jss.v028.i05.
